# Awareness of the Link Between Human Papilloma Virus Infection and Head and Neck Cancer Among the General Population and Practitioners: A Literature Review

**DOI:** 10.3390/cancers16213556

**Published:** 2024-10-22

**Authors:** Alicia Tosoni, Linda Galvani, Vincenzo Di Nunno, Lidia Gatto, Stefania Bartolini, Marta Aprile, Elisa D’Angelo, Ernesto Pasquini, Anna Maria Baietti, Enrico Franceschi

**Affiliations:** 1 Nervous System Medical Oncology Department, IRCCS Istituto delle Scienze Neurologiche di Bologna, 40139 Bologna, Italy; vincenzo.dinunno@isnb.it (V.D.N.); lidia.gatto@ausl.bologna.it (L.G.); stefania.bartolini@isnb.it (S.B.); e.franceschi@isnb.it (E.F.); 2 Department of Experimental, Diagnostic & Specialty Medicine, University of Bologna, 40138 Bologna, Italy; linda.galvani@studio.unibo.it (L.G.); marta.aprile@studio.unibo.it (M.A.); 3 Radiation Oncology Department, Bellaria Hospital, AUSL Bologna, 40139 Bologna, Italy; elisa.dangelo@ausl.bologna.it; 4 ENT Unit, Bellaria Hospital, AUSL Bologna, 40139 Bologna, Italy; ernesto.pasquini@ausl.bologna.it; 5 Facial Surgery and Dentistry Network—Trauma and Severe Disabilities Path Department, AUSL Bologna, 40139 Bologna, Italy; annamaria.baietti@ausl.bologna.it

**Keywords:** human papilloma virus, HPV, head and neck cancer, awareness

## Abstract

**Background:** Human papilloma virus (HPV) infection is responsible for 4.5% of cancers worldwide, i.e., for around 630,000 new cases per year. Around 38,000 cases per year of HPV-related cancers arise in the head and neck region, of which 21,000 cases are oropharyngeal squamous cell carcinomas (OPSCCs), with an increasing frequency in high-income countries. In our work, we aimed to collect the available evidence about the awareness of the relation between HPV infection and head and neck cancer among patients and practitioners. **Methods:** This review was based on all available prospective and retrospective studies, case reports, and review articles published up to May 2024 in PubMed. The search keywords used included “human papilloma virus” plus “head and neck cancer”, “awareness”, “infection”, “vaccination”, “awareness in patients and practitioners”, “oropharyngeal squamous cell carcinoma”, “HPV16-18”, “HPV awareness”, “cervical cancer”. **Results:** In the U.S.A., with more than 150.00 participants interviewed through different studies, the identification of HPV infection as a risk factor for head and neck cancer (HNC) was made by only 12–39% of the participants. Similar conclusions were drawn from European studies in which only 10–52% of the participants recognized HPV as a risk factor for HNC. HPV-related HNC awareness in health care providers and medical/dental students showed a high variability among studies, being otolaryngology and dental sciences specialists the most conscious in comparison to general practitioners, who demonstrated a low level of awareness. **Conclusions:** There is a dangerous lack of awareness about HPV infection and its potential role in HNC among both general populations and health care practitioners. A great effort should be made to increase awareness in both practitioners and the general population on this topic, also aiming to increase the HPV vaccination coverage.

## Simple Summary

Human papilloma virus infection is responsible for 4.5% of cancers worldwide. Around 38,000 cases per year of HPV-related cancers arise in the head and neck region, of which 21,000 cases are oropharyngeal squamous cell carcinomas (OPSCCs), with an increasing frequency in high-income countries. There is a dangerous lack in awareness about HPV infection and its potential role in HNC among both general populations and health care practitioners. In our work, we aimed to collect the available evidence about the awareness of the relation between HPV infection and head and neck cancer among patients and practitioners. A great effort should be made to increase both practitioners and general population’s awareness on this topic, also aiming to increase HPV vaccination coverage.

## 1. Introduction

HPV infection is responsible for 4.5% of cancers worldwide, with an HPV-related cancer incidence of 620,000 cases in women and 70,000 in men in 2019 [1]. Cervical cancer is the most common HPV-related cancer and is also the fourth most common female cancer, accounting for 600,000 new cases annually. Importantly, 83% of all new diagnoses are reported in low-, middle-low-, and middle-income countries [1].

Other HPV-related types of cancer arise in the anogenital and in the head and neck region. Around 38,000 cases per year of HPV-related cancers arise in the head and neck region, of which 21,000 cases are oropharyngeal squamous cell carcinomas (OPSCCs), with an increasing frequency in high-income countries [2]. The reduction in smoking habits and alcohol consumption, the risk factors historically related to head and neck cancer (HNC), has led to a decreasing incidence of HPV-negative HNC in developed countries in the last two decades. Conversely, a shift in HNC epidemiology has been observed, with a rise in HPV-related OPSCCs in the same period [3]. In particular, HPV is now related to 71% of OPSCC cases in the U.S.A. and 51.8% in the U.K. [4,5]. The incidence of HPV-positive OPSCCs in Italy, among OPSCC cases, increased from 16.7% in 2000–2006 to 46.1% in 2013–2018 [6]. The HPV-AHEAD study, a retrospective cohort study conducted in Italy taking account of 696 HNC patients, showed a percentage of 44.4% of HPV-related cases [7]. HPV-positive and HPV-negative OPSCCs also differ in clinical outcomes, with the first one appearing to be related to better overall survival and progression-free survival [8,9]. The distinct molecular profiles, tumor characteristics, and outcomes have led the American Joint Committee on Cancer (AJCC) staging system to separate HPV-positive and HPV-negative OPSCCs [10]. Unfortunately, no screening methods for the early detection of HPV-associated OPSCC are currently available; therefore, a widespread prevention can be achieved only through large-scale vaccination programs.

Prophylactic HPV vaccination is known to prevent HPV-related cervical cancer and is expected to be also effective in preventing HPV-related HNC, as many countries already administer it both in boys and in girls [11,12,13]. HPV types 16, 18, 31, 33, 35, 39, 45, 51, 52, 56, 58, and 59 are classified as carcinogenic, and HPV68 as probably carcinogenic, and they are referred to as high-risk types [14]. There are currently three available HPV-vaccines: Cervarix (GlaxoSmithKline), a bivalent HPV vaccine that targets HPV16 and 18; Gardasil (Merck Inc.), a quadrivalent HPV vaccine that targets HPV16/18 and also the low-risk types HPV6 and 11 linked to genital warts; and Gardasil 9 (Merck Inc.), a nine-valent vaccine that targets HPV6/11/16/18/31/33/45/52/58 [3]. While the link between HPV and cervical cancer is increasingly acknowledged in the general population, even if a complete awareness is still lacking, and there are still some uncertainties even among practitioners in recommending the vaccination, the link between HPV and HNC is often ignored even among health specialists, and the role of HPV infection in carcinogenesis is underestimated [15]. The same trend of low awareness, as intended for HPV to have a causative role in these types of cancer, has been observed for the link between HPV and vulvar cancer [16,17,18].

In our work, we aimed to collect the available evidence about the awareness of the relation between HPV infection and HNC among patients and practitioners.

## 2. Materials and Methods

**Figure 1:**
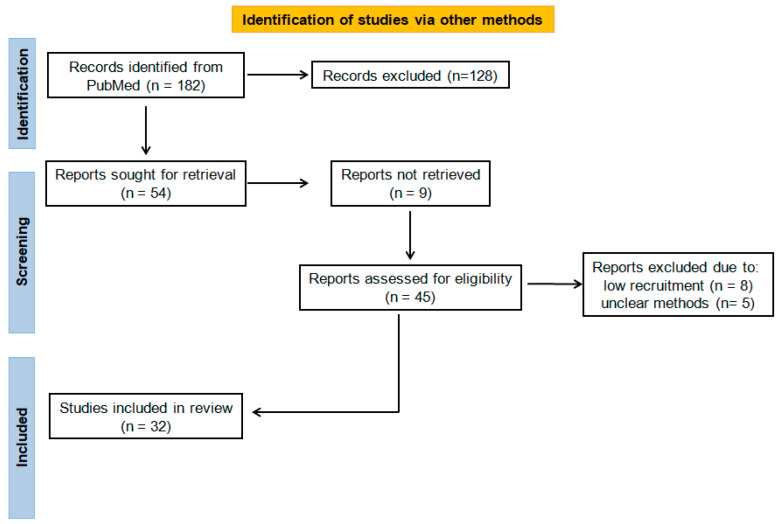
Flowchart of the selected studies.

This review was based on all available prospective and retrospective studies, case reports, and review articles published from March 2008 up to May 2024 in PubMed. The search keywords used included “human papilloma virus” plus “head and neck cancer”, “awareness”, “infection”, “vaccination”, “awareness in patients and practitioners”, “oropharyngeal squamous cell carcinoma”, “HPV16-18”, “HPV awareness”, “cervical cancer”. We then subdivided the collected works in sections, based on whether HPV awareness was referred to the general population, health care practitioners, or patients (see Figure 1 and Supplementary Material).

## 3. Results

### 3.1. General Population

Public awareness of HPV is lacking worldwide, particularly in regard to the relation between HPV and HNC.

Eighteen studies were conducted from 2014 to 2023 both in the U.S.A. and in Europe to assess knowledge and awareness of HPV infection relating to HNC among different classes of participants to more precisely target screening and education efforts in the future [19,20,21,22,23,24,25,26,27,28,29,30,31,32,33,34,35,36]. The mentioned studies, even if conducted with different types of questionnaires administered to different classes of subjects, seemed not to show a difference in HPV-HNC awareness over the years. Up to date, no data are available on the HPV-HNC relation awareness in Africa, Asia, and Oceania.

#### 3.1.1. General U.S. Population

In the U.S.A., with more than 150.00 participants interviewed through different studies, the identification of HPV infection as a risk factor for HNC was made by only 12–39% of the participants (Table 1). In particular, Berger et al. [19] aimed to assess the awareness of HPV infection as a cause of HNC by performing a survey at a cancer screening event. Among 308 patients, including 196 women and 112 men, 40% of the respondents reported having heard of HPV, while only 28% of them knew that HPV could cause HNC. Women were found to be more than two times more likely than men to have heard of HPV. Among people who answered of having heard of HPV, the majority was aware that HPV was sexually transmitted. Again, Luryi et al. [22] conducted an online survey on 2126 randomly selected adults in the United States, revealing that only 12.8% of them were aware of the association between HPV infection and OPSCC, whereas 70% of the respondents were aware of the availability of HPV vaccination. Similarly, a 23-question survey was administered to a cohort composed of 319 civilians and 158 military officers, randomly chosen as participants at various local shopping malls and at Maxwell Air Force Base in 2012. The survey showed that 75% of the civilians and 49% of the militaries were not aware that HPV can cause HNC. Furthermore, the majority of the participants did not know that both males and females are eligible for HPV vaccination [23]. Additionally, the link between sexual orientation and HPV-related HNC awareness was examined by collecting data from the Health Information National Trends Survey of 10,859 adult participants between 2017 and 2019. The study statistically evaluated the differences in HPV-related HNC awareness between sexual minorities and heterosexual participants, finding a cumulative awareness of only 19% among the respondents. No significant differences in knowledge were observed between the sexual minority group and the heterosexual group [20]. Another work by Franca et al. [21] investigated HPV awareness among a younger population of 862 college students by asking them if they knew whether HPV was causally linked to anogenital cancer and HNC. About 70% of the participants were aware of the link between HPV and cervical cancer, while a lower percentage of awareness was observed for the link with cancer in other anogenital sites and HNC. In another cohort of young patients aged from 12 to 24 years and their parents at a pediatric clinic, more than 78% of the participants declared that they would be more likely to receive the HPV vaccine if given strong evidence about its prevention of HNC [25]. Regarding vaccination, an article by Dundar et al. [24] aimed to collect, throughout an anonymous survey, the propensity of parents of children between the age of 9 and the age of 18 towards HPV vaccination and their awareness about the association between HPV and HNC. Only 12% of the parents had heard about HPV-related HNC. The main concerns about vaccination were related to effectiveness and side effects, and the lack of knowledge about HPV-related cancers was a significant deterrent to vaccination. Additionally, in a recent survey of a high-risk population conducted at a Drag Racing event, less than one-third of the participants were aware that HPV infection can cause HNC, more than 40% of them believed that HPV was the same as HIV, and only 25.1% of them received information about HPV infection and prevention from a health care practitioner. The participants that thought that the number of sexual partners was not related to HPV infection were more likely to have low knowledge scores and were 33 times more likely to have a lower awareness of the link between HPV and HNC (OR = 33.27; 95% CI: 16.34, 67.74) [26].

#### 3.1.2. General European Population

Similar conclusions on HPV awareness were drawn from European studies (Table 2). A population-based survey was conducted among 1095 adults from a representative sample of the German population with a specific emphasis on awareness of HPV, its transmission, and symptoms indicative of OPSCC [27]. The study demonstrated that 63.8% of the participants had never heard of HPV, and only 14.2% of them recognized HPV as a risk factor for HNC, independently of gender or family status. In addition, 44.9% of the subjects knew that a preventive vaccine against HPV exists, with women being significantly more aware of HPV (34.2% vs. 22.8%) and vaccination (56.4% vs. 32.7%) compared to men. Younger individuals (age group 25–34 years) were significantly more aware of HPV, likely because they were offered HPV vaccination. Data from an online cross-sectional survey collected in the Netherlands [31] revealed that only 30.6% of the participants had heard of HPV, with a knowledge gap among males, people older than 65 years, people with a low education level, and smokers. Among those who had heard about HPV, less than 30% were aware of the association between HPV and HNC. Less than half of the participants knew about the existence of HPV vaccines. Furthermore, a survey distributed to 508 women in Spain showed that less than a half of them identified HPV as a possible cause of HNC. Education, sexual behavior, and employment status were associated with a poorer level of awareness [35]. In Italy, two different surveys were performed. The first study [29] administered a 17-item questionnaire related to HPV to a group of 934 patients at their first access to health centers for uroandrological issues, comparing the results with those of an age-comparable group of 172 nurses. Overall, about 80% of them indicated not being aware that HPV infection can cause HNC. HPV-related HNC knowledge was recorded for 7.3% of the patients compared to 22.1% of the nurses, whereas HPV vaccine knowledge was found for 29.3% and 75.6% of the participants, respectively. Female gender and educational status were statistically associated with an increase in HPV awareness. The second Italian study explored women’s awareness in 446 women attending primary care centers, showing that only 34.7% of the participants knew that HNC can be related to HPV. Less than 25% of them had received HPV vaccination, but more than 60% believed that HPV vaccination is effective to prevent HNC. Similarly, a Polish study [33] that collected data from 201 women who underwent a cervix high-risk HPV DNA testing, showed a statistically significant link between age and HPV oncogenic potential awareness, with women aged over 43 years being more conscious of HPV-related lesions. With the aim to investigate the propensity towards HPV vaccination among adults in Poland, a cross-sectional survey by Pinkas et al. [34] showed that less than 25% of the participants chose vaccination as an HPV infection prevention method, and only 48.1% of them showed propensity towards HPV vaccination. Observations among a younger population demonstrated similar results. In particular, the awareness about HPV infection and its oncogenic role was investigated among Poland university students using a 22-question survey [32]. A group of 1710 students participated, including both medical and non-medical students. Among the non-medical students, 60% had never heard about HPV in comparison to 96% of the medical students. Only 45% of the non-medical students knew about the existence of an HPV vaccine, in contrast to more than 70% of the medical students. The oncogenic potential of HPV was known by 81% of the medical students and only by 56% of the non-medical students. Around half of the participants from both groups did not know about the risk of developing oropharyngeal cancer. Again, a cross-sectional survey was conducted among 1415 United Kingdom university students (495 men and 920 women), aged 18–25 years. Among them, 70% had heard of HNC, but only 25% were aware that it could be related to HPV. Again, women more likely acknowledged this link. Similarly, a questionnaire-based survey about HPV, HNC, and HPV vaccination was carried out among 1550 Irish university students [28]. More than half of them were unaware of how HPV can be transmitted, and 45% had never heard about HPV. More than 80% of them were completely unaware of the link between HNC and HPV.

### 3.2. Health Care Providers, Medical and Dental Practitioners, Medical and Dental Students

Medical professionals and professional students could play an important role in encouraging patients to undergo HPV vaccination. Several studies investigated HPV knowledge and awareness in this setting.

Some studies conducted in different geographical areas evaluated HPV-related HNC awareness in medical student populations.

Nevada university administered a 25-question online survey to professionals and medical students. Among the latest, the same questionnaire was administered before and after a vaccine workshop. One-third of the participants were aware of the link between HPV and HNC, and most of them believed that the HPV vaccination should be mandatory. The propensity to believe in mandatory vaccination was significant associated with a full vaccination status (OR = 2.63), awareness of the link between HPV and oropharyngeal cancer (OR = 1.85), and female sex (OR = 1.64) [37]. Similarly, among 617 New York State medical students, 99% correctly identified the link between HPV infection and cervical cancer, whereas fewer knew about the link with HNC (47.2%) [38]. A similar situation was showed in a Jordan survey conducted among 1198 medical students, demonstrating that whereas all the participants had heard of HPV, only 21% identified the association between HPV and HNC. Additionally, 34% of the students were not aware of the availability of HPV vaccines. The majority of the students (92.0%) reported that the university courses were their major source of knowledge about HPV. University courses were reported as the major source of HPV awareness [39].

Other studies evaluated HPV-related HNC awareness among dental students.

Sallam et al. conducted a parallel paper-based survey in Jordan pre-clinical and clinical dental students, interns, and postgraduate maxillofacial residents, demonstrating a satisfying knowledge about the role of HPV in HNC that was recognized in most of the clinical group participants and in 82% the pre-clinical group participants, albeit with gaps in the awareness of HPV vaccine availability, which was known by 44% of the pre-clinical group participants and 37% of the clinical group ones [40]. On the contrary, in a group of 500 undergraduate dental students interviewed in Saudi Arabia, HPV, HPV vaccination, and HNC knowledge was low, with 62% of the students having heard of HPV, 57% of HPV vaccination, and those showing higher HPV awareness scores being more willing to take the HPV vaccine (66%) [41]. Similarly, a group of 197 Romanian dental students underwent a 22-question survey, which showed a lack of awareness about the potential prevention of oral cancer through anti-HPV vaccination in 39.7% of the fifth year participants [42]. A better recognition of the role of HPV in HNC was showed in 158 Spanish dental students, most of which knew that HPV infection can be linked to HNC (75%) [43].

Unfortunately, only few studies examined the knowledge of health care professionals about HPV-related HNC. This issue was investigated in 404 Polish physicians, including 192 general practitioners, 68 trainees, and 144 otorhinolaryngologists. Among the latest, 86.8% had contact with HNC patients, while most of the general practitioners did not. Surprisingly, about 7% of the otolaryngologists and 20% of the general practitioners had never heard about HPV in oropharyngeal diseases. Interestingly, while 100% of the participants had heard about the role of vaccines in the reduction of the incidence of cervical cancer, only 29% of them had heard about the possible role of vaccination in preventing HNC [44].

Similarly, as regards pediatricians, an 18-question survey was administered to 116 members of the Louisiana Chapter of the American Academy of Pediatrics. Most of them routinely recommended the HPV vaccine, but 45.9% were not aware of the link between HNC and HPV [45]. Alarming data also emerged from a self-administered survey among the non-clinical staff at community-based HIV/AIDS Service Organizations in South Carolina and Texas, reporting that 32% of the participants were not aware about the HPV link with HNC, with over a half of them being unsure about the effectiveness of vaccination to prevent HNC [46].

### 3.3. Patients and High-Risk Population

Even less data are available regarding HPV-related cancer patients and caregivers. In a cross-sectional online survey carried out among 200 HPV-related cancer survivors, only about one-third of the participants knew that their cancer was HPV-related, and 56.8% indicated HPV vaccination as safe. The participants who were aware that their cancer was HPV-related were more likely to vaccinate their children, and the participants who believed in vaccination safety were more willing to recommend it, to be a mentor for others with HPV-related cancers, and to act as advocates for vaccination [47].

## 4. Discussion

Our report demonstrates limited worldwide awareness of the link between HPV and HNC. Unfortunately, there is a great heterogeneity between the collected studies, for both those referred to the general population and those referred to practitioners.

However, even if a direct comparison cannot be made, the data seem to show no difference in HPV-related HNC awareness in the general population in Europe (12–39%) and the U.S.A. (10–52%, see Table 1).

Our review shows how HPV infection knowledge and HPV-related HNC awareness may vary profoundly depending on the education level [23,31]. A work by Chen et al. observed the variations in mortality rates for patients with HNC between 1993 and 2007, demonstrating that those with a schooling time greater than 12 years presented a decreased mortality irrespective of race and gender, which could reflect different smoking habits and sexual behaviors among people of different educational levels [48]. Women seems to be more aware of HPV oncogenic potential, probably due to the widespread of the screening programs for cervical cancer [19,36].

A sufficient knowledge on HPV-related HNC among general practitioners and dentists is critical, since early diagnosis may play an important role in reducing the mortality from the disease.

In line with this, a difference in awareness between the general population and health providers seems to be present, even if among practitioners, there is a high variability [44].

When investigating providers’ awareness by specialty, otolaryngology and dental sciences specialists were the most conscious about the link between HPV and HNC, while awareness was low among general practitioners [44]. This may impact the general population’s knowledge, since a smaller fraction of it would present to these specialty providers. An increase in awareness among general practitioners should be encouraged to reach more individuals, especially those from high-risk populations.

Analyzing the results on the level of knowledge of future medical doctors and dentists, a better awareness seemed to be present in dental students in comparison to medical students (60–97% versus 21–47%, see Table 3). Interestingly, among medical and dental students, those in the final years were more in favor of vaccination programs and were more aware of HPV infection and its link with HNC [40].

All the collected evidence shows how increasing both general population’s knowledge and practitioners’ awareness of HPV-associated HNC would provide a more thorough understanding of this topic and a greater propensity towards vaccination [24,45].

A scarce awareness in the general population could also represent an obstacle to seeking care, while a scarce knowledge among general practitioners could lead to misdiagnoses or to delays in diagnosis. In the last two decades, an increase in publications and studies about this topic has been observed, addressing a need for a greater understanding of the issue. On the other hand, data on HPV-related HNC patients and caregivers are scarce, and a great effort has to be made to understand the level of awareness in this setting, with the aim to develop information programs and campaigns to expand patients’ and collective awareness.

Regarding vaccination, 60% of WHO member states had added the HPV vaccine into their vaccinal schedule up to March 2022; most of these countries are high- and upper-middle-income states. Many of the most populated nations have not introduced HPV vaccination yet into their immunization schedule, leading to a low global coverage of 12% for two doses in females [49]. Unfortunately, the HPV vaccinal coverage among males is further lower than among females, highlighting an important lack of awareness of the prophylactic role of HPV vaccination for preventing HNC and other non-gynecological cancers, such as penile cancer. Current evidence suggests the increasing necessity for a broader and gender-neutral HPV vaccination program [11,50].

An urgent need for an increase in vaccinal coverage should be addressed by health care providers encouraging educational interventions, such as informative campaigns about the role of HPV in both cervical and non-cervical cancers, as well as the role of vaccination in preventing them.

In conclusion, a great disparity in awareness regarding HPV and HPV infection’s causal effect on HNC was observed. A lack in awareness exists for both the general population as well as health care providers. The lack in knowledge among health care providers can have a negative impact on vaccination coverage, since many works pointed out that thorough information about HPV infection’s potential link to HNC may convince people to get vaccinated and to vaccinate their children [24]. A great effort should be made to increase awareness in both practitioners and the general population of HPV infection and its related oncogenic potential.

## 5. Strengths and Limitations

This review reported and analyzed a wide range of studies from different geographical regions, providing a broad perspective on HPV awareness levels among both the general population and health care providers. Given the increasing incidence of HPV-related cancers, particularly OPSCCs, this review addressed an important topic, i.e., the awareness of the link between HPV infection and HNC. Limitations of the review are due to the great heterogeneity between all the collected studies, with different surveys and data collection methods, as well as cultural factors and differences in health care access.

## 6. Conclusions

There is a dangerous lack in awareness about HPV infection and its potential role in HNC among both general populations and health care practitioners. A great effort should be made to increase awareness in both practitioners and the general population on this topic, also aiming to increase the HPV vaccination coverage. Vaccination awareness campaigns should therefore focus on increasing awareness of the risks associated with HPV infection but also on developing more specific interventions targeting males.

## Figures and Tables

**Table 1:** HPV-HNC awareness in the U.S. general population.

Author	Year	No. Prt	Median Age	HPV-HNC Awareness #
Berger [16]	2018	308	54	28%
Dougherty [20]	2023	10,859	56	19%
Franca [21]	2023	862	20	39%
Luryi [22]	2014	2126	42	13%
Williams [23]	2015	477	NR	36%
Dundar [24]	2022	150	NR	12%
Kram [25]	2015	119	12	20%
Osazuwa-Peters [26]	2015	303	48	30%

No., number; prt, participants, NR, not reported; HPV, human papilloma virus; HNC, head and neck cancer; # percentage of subjects interviewed knowing about the association between HPV and HNC.

**Table 2:** HPV-HNC awareness in the European general population.

Author	State	Year	No.	Median Age	HPV-HNC Awareness #
Sharma et al. [27]	Germany	2022	1095	NR	16%
Kavanagh et al. [28]	Ireland	2018	1550	24	16%
Capogrosso et al. [29]	Italy	2015	934	40	10%
Paduano et al. [30]	Italy	2023	446 *	39	35%
Verhees et al. [31]	Netherland	2021	1044	NR	27%
Jeruzal-Świątecka et al. [32]	Poland	2020	1710	25	52%
Wencel-Wawrze ’nczyk et al. [33]	Poland	2022	201 *	NR	NR
Pinkas et al. [34]	Poland	2022	1082	44	32%
Lorenzo Pouso et al. [35]	Spain	2022	409 *	44	49%
Dodd et al. [36]	UK	2021	1415	20	25%

* Only women; No., number; NR, not reported; HPV, human papilloma virus; HNC. head and neck cancer; # percentage of subjects interviewed knowing about the association between HPV and HNC.

**Table 3:** HPV and HPV-HNC awareness in medical students, dental students, and health care providers.

Author	State	Year	No.	Population	HPV-HNC Awareness #
**Medical students**					
Evans et al. [37]	USA	2020	333	Medical students and MDs	33.6%
Laitman et al. [38]	USA	2018	617	Medical students	47.2%
Sallam et al. [39]	Jordan	2022	1198	Medical students	21%
**Dental students**					
Lorenzo-Pouso et al. [43]	Spain	2019	158	Dental students	75%
Murariu et al. [42]	Romania	2022	197	Dental students	60%
Sallam et al. [40]	Jordan	2019	376	Dental students	Preclinical group 83%
					Clinical group 97%
Farsi et al. [41]	Saudi Arabia	2020	500	Dental students	NR
**Health providers**					
Jackowska et al. [44]	Poland	2015	404	ENT specialists	93%
				General practitioners	80%
				Trainees	90%
Mehta et al. [45]	USA	2017	116	MD pediatricians	46%
Tomar et al. [46]	USA	2020	51	Non-clinical staff	32%

No., number; NR, not reported; HPV, human papilloma virus; HNC, head and neck cancer; MDs, medical doctors; ENT, otolaryngologists; # percentage of subjects interviewed knowing about the association between HPV and HNC.

## Supplementary Materials

The following supporting information can be downloaded at: https://www.mdpi.com/article/10.3390/cancers16213556/s1, Preferred Reporting Items for Systematic reviews and Meta-Analyses extension for Scoping Reviews (PRISMA-ScR) Checklist [51].
